# A web app-based music intervention reduces experimental thermal pain: A randomized trial on preferred versus least-liked music style

**DOI:** 10.3389/fpain.2022.1055259

**Published:** 2023-01-16

**Authors:** Orelle Soyeux, Serge Marchand

**Affiliations:** ^1^ International Laboratory for Brain, Music and Sound Research (BRAMS), University of Montreal, McGill University, Montreal, QC, Canada; ^2^ Department of Psychology, University of Montreal, Montreal, QC, Canada; ^3^ Research Centre, Sherbrooke's University Hospital, Sherbrooke, QC, Canada; ^4^ Faculty of Medicine and Health Sciences, University of Sherbrooke, Sherbrooke, QC, Canada

**Keywords:** music, pain, digital therapeutics, experimental pain in humans, adult, endogenous pain modulation, conditioned pain modulation

## Abstract

Digital technologies are increasingly being used to strengthen national health systems. Music is used as a management technique for pain. The objective of this study is to demonstrate the effects of a web app-based music intervention on pain. The participants were healthy adults and underwent three conditions: Conditioned Pain Modulation (CPM), Most-Liked Music (MLM) and Least-Liked Music (LLM). The music used is MUSIC CARE©, a web app-based personalized musical intervention (“U” Sequence based on a musical composition algorithm). Thermal pain was measured before starting the 20-min music intervention and after three time points for each music condition: 2.20, 11.30, and 20 min. Mean pain perceptions were significantly reduced under both LLM and MLM conditions. Pain decrease was more important under MLM condition than LLM condition at 2.20 min with a mean difference between both conditions of 9.7 (±3.9) (*p* = 0.0195) and at 11.30 min [9.2 (±3.3), *p* = 0.0099]. LLM is correlated with CPM but not MLM, suggesting different mechanisms between LLM and MLM. Musical intervention, a simple method of application, fits perfectly into a multidisciplinary global approach and helps to treat the pain and anxiety disorders of participants.

**Clinical trial registration:** [https://clinicaltrials.gov/ct2/show/NCT04862832], ClinicalTrials.gov [NCT04862832].

## Introduction

Music has been reported as a management technique of acute and chronic pain since 1960 (1) and is nowadays widely used as an alternative or complementary treatment to reduce patient pain (2). Music is easy to implement in clinical contexts as it is safe, non-invasive, and inexpensive (3, 4). Multiple clinical environments can be found using music today (5, 6) for conditions such as childbirth (7–10), resuscitation (11), cardiac surgery (12, 13), cancer (14, 15), in cardiology, during a catheter installation (16), or cataract surgery (17).

Converging evidence suggests that music is indeed beneficial for different types of pain (13, 14, 18, 19), in addition to psychological distress, ranging from smaller-scale mood improvements to anxiety disorders (20). Music can also improve the management of chronic pain conditions, such as cancer by reducing pain and its associated components of anxiety, depression, and quality of life (21).

There are different music procedures used to reduce pain (22), especially one based on music in a medical context (23). In the treatment of pain, the most widely used music is relaxing (5), even if no consensus was reached to indicate a difference between relaxing and stimulating music's ability to reduce pain.

A multitude of endogenous mechanisms can modulate pain perception. Music involves different inputs such as sensory, cognitive, or emotional (24), which, according to the Neuromatrix theory of pain (25) can modulate the final pain perception. Music-induced analgesia could be explained by a change of perception such as distraction (26). Another potential mechanism is the recruitment of Conditioned Pain Modulation (CPM). CPM is based on the recruitment of diffuse noxious inhibitory control (DNIC) from different structures in the brainstem (e.g., periaqueductal gray, nucleus raphe Magnus) following a localized nociceptive stimulation (27). However, CPM is also influenced by descending higher center activities (28). Music is suggested to increase the efficacy of descending mechanisms like conditioned pain modulation (CPM) (29). Brain imaging studies reported that listening to music activate spinal and supraspinal regions known to be involved in endogenous descending pain modulation (30).

Perceived pleasantness has also been suggested to play a role in the analgesic potential of music. Pleasant music according to the participant is superior to unpleasant music or silence in decreasing experimental pain (31). Allowing the patient to choose the style of music adds to pain relief and adds a sense of control over pain (5, 6, 32–34). Paying attention to personal musical preferences and cultural background are among the main characteristics of a successful musical intervention (35, 36). However, in many studies evaluating the effect of music on pain, there is a lack of details of the musical choice (33). It was also reported that pain was even more reduced when participants were selecting preferred music from a list given by the researcher (5). Recent technological developments now enable patients or caregivers to control the use of music-based interventions using hand-held devices. Silence condition is frequently used as a control condition for music, as sensory, cognitive or emotional inputs are limited during this condition.

Studying the effect of music-based interventions in medical contexts is complex and requests strong methodology. Discussion with researchers in this field suggested that the main methodological research challenges relate to treatment, outcomes, research designs, and implementation (37). According to a systemic review, music sessions should last between 20 and 60 min and consist principally of harmonic variations (38). There is a growing interest to design and implement new and cost-effective online treatments using technological advances. The Ministries of Health of the WHO European Region are increasingly investing in Digital Therapeutics (DTx). They are helping overcome barriers to the adoption of DTx to strengthen health systems and to explore ways to accelerate DTx for public health. Digital health technologies can improve access to health services, lower costs, improve the quality of care and increase the efficiency of health systems. They offer ways to manage personal health, with a focus on disease prevention rather than just treatment (39).

Based on these recent scientific recommendations, MUSIC CARE©, a web app-based personalized music intervention, has developed a “U” Sequence based on a musical composition algorithm. The music sequence can last from 20 to 60 min and is divided into several phases that gradually enable the patient to lower their pain and anxiety levels in line with the “U” Sequence technique (11, 40, 41). Previous studies had confirmed the effectiveness of this web app-based music intervention in reducing pain and/or anxiety in patients with a variety of conditions (2, 42, 43).

The principal objective of this study was therefore to describe the effects of a web app-based music intervention on the modulation of pain and the difference between the most-liked music (MLM) and least-liked music (LLM) conditions. A secondary objective was to compare the effect of CPM with MLM and LLM on pain perception. Our hypothesis was that most-liked music will be superior to least-liked music in reducing pain perception. Our second hypothesis was that the pain relief during the music interventions would be correlated with CPM, suggesting comparable mechanisms.

## Materials and methods

### Study design

A randomized, multi-center, open-label, controlled, crossover clinical trial was conducted in four centers: Sherbrooke University Hospital Centre, Sherbrooke University, the campus of Bishop's University and Cégep Champlain. Participants were recruited in these 4 centers, but the procedures were conducted at Sherbrooke University Hospital Centre by two research assistants. This study was composed of 3 experimental sessions on 3 consecutive days: on day 1 CPM was tested in all participants for a baseline endogenous pain inhibition measurement. All subjects then entered a randomized crossover part of the study with MLM or LLM on day 2 and day 3.

### Participants

**Figure 1:**
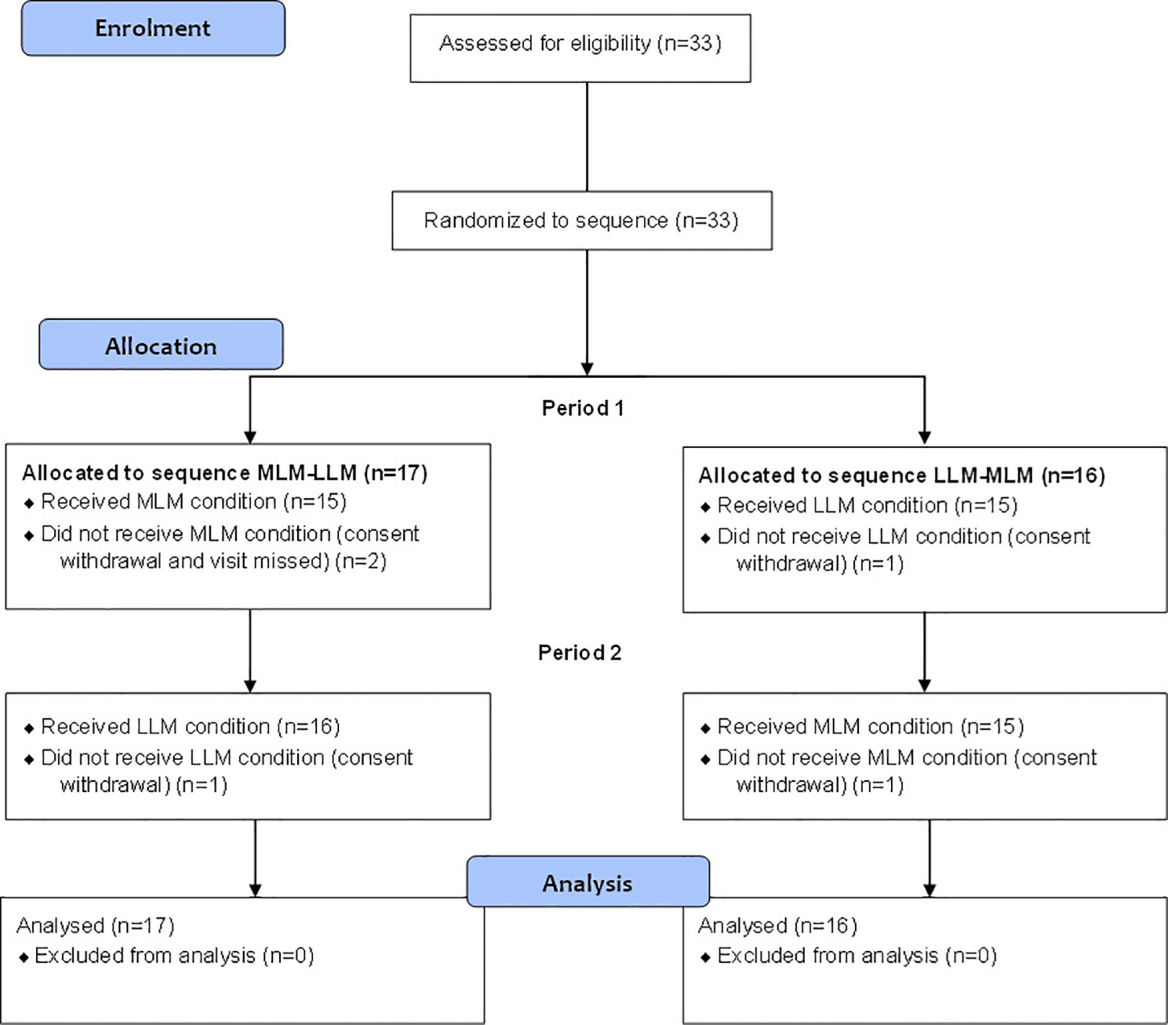
Participant flow chart following consolidated standards of reporting trials guidelines for MLM-LLM and LLM-MLM randomized arms.

Thirty-three healthy adults [20 women and 13 men mean age of 23.5 years (±4,9)] participated in this study. Participants were excluded if they were musicians with knowledge of music theory, diagnosed and taking medication for chronic pain, skin problems, psychological or neurological pathologies. The protocol was approved by the ethics committee of *Centre Hospitalier Universitaire de Sherbrooke* and informed consent was obtained from all participants. The verbal and written instructions including the questionnaires were presented in French or English at the choice of the participant. The patient flow chart is presented in Figure 1.

### Web app-based music intervention

**Figure 2:**
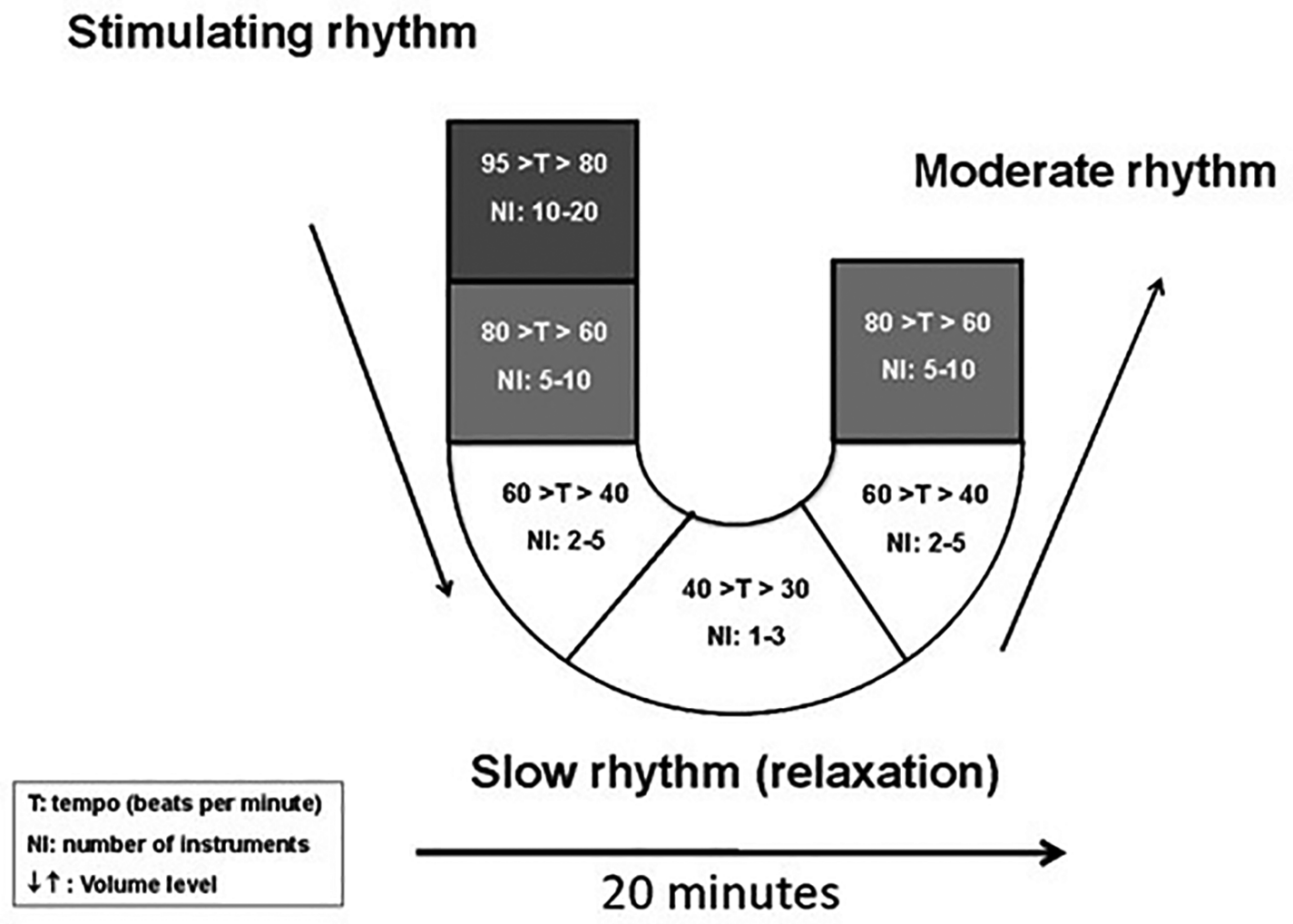
The “U” sequence. The musical sequence of 20 min is divided into 6 different musical pieces at 3–4 min each.

The web app-based music intervention was administered using headphones *via* a tablet-based application called *MUSIC CARE©*. The *MUSIC CARE©* app is a receptive music intervention and utilizes the “U” sequence (Figure 2) designed to gradually relax the listener (41–43).

It is implemented using a musical sequence of 20 min that was divided into 6 different musical pieces at 3–4 min each. The first five sections are in minor mode where the first one starts with stimulating musical rhythm 80–95 beats per minute (bpm). Then, the remaining four sub-pieces are presented in a blended fashion in an attempt for the patient to gradually fall into a relaxed state *via* a gradual reduction in musical tempo (40–80 bpm), orchestral size, frequencies, and volume (descending arms of the “U”) followed by a phase of maximum relaxation (downward phase of the “U”). The last section is in major mode which corresponds to a phase that gradually returns to baseline dynamics (ascending arms of the “U”). This is thought to induce a catharsis. This construction is hypothesized to allow a mirror effect with the patient's emotions throughout the sequence. This is similar to the iso principle of music therapy (44) that describes the process of alteration of the patient's state by music. The minor mode validates the patient's suffering (negative emotions) and then the relaxation phase calms the patients, and finally the major mode incites positive emotions. In 1936 it was shown that the minor mode is known to be related to negative emotions while the major mode is related to positive emotions (45).

Thirty musical sequences were available (classical, folk, jazz, reggae and traditional music from South America, Caledonia, Asia, India or the Middle East) (Table 1), allowing for a personalized choice for the subjects. The music sequences are all 20 min, instrumental, professionally recorded in the studio and composed specifically for the MUSIC CARE© application and are thus unfamiliar to participants. The participants of this group listened, with headphones (QuietComfort® 25 Acoustic Noise Cancelling® headphones, Framingham, Massachusetts, Bose Corporation) plugged to a tablet (Samsung Galaxy Tab, 2013, 3 Lite 7.0, Suwon, South Korea, Samsung Electronics Co., Ltd.) to 30 s samples of the available sequences and rated them on a 0–10 scale in which 0 is “I do not want to listen to this one” and 10: “I really want to listen to this one.” The order of the 30 samples was randomized for each participant. The highest rating was selected for the MLM condition and the lowest was for the LLM condition.

**Table 1:** Number of participants by musical style choices for most-liked music (MLM) and least-liked music (MLM).

Style	Cuban	Flamenco	Celtic	Asian	African	Oriental	Indian	Reggae 1	Reggae 2	South American	Afro beat	Guitar ballad	Blues
Chosen as MLM	0	3	2	0	2	1	0	2	0	1	0	0	2
LLM	1	0	0	3	1	3	1	0	1	1	0	1	0
	Electro jazz	Jazz ballad	Folk guitar		Rock guitar	Piano	Accordion	Classical music 1 (Classical)	Classical music 2 (Classical)	Classical music 3 (Romantic)	Classical music 4 (Romantic)	Classical music 5 (baroque)	Film music
Chosen as MLM	0	0	2		2	0	2	2	2	3	4	1	2
LLM	13	1	2		1	0	1	1	0	0	0	0	0

### Thermal stimulation and conditioned pain modulation (CPM)

CPM was measured the first day in all subjects to have a baseline of the efficacy of endogenous pain modulation as previously describe (46). Since CPM is variable amongst healthy subjects and patients (47, 48), this baseline permitted to test for a correlation in pain changes between CPM and music sessions that could suggest comparable mechanisms.

The CPM paradigm to study the efficacy of inhibitory mechanisms is obtained by calculating the difference in pain levels elicited by the test stimulus (TS) before and after the conditioning stimulus (CS) (46, 48). The TS was generated by a 3 cm^2^ thermode (TSA II, NeuroSensory Analyzer, Medoc Instruments, North Carolina, USA) applied on the non-dominant forearm of each participant at a predetermined, individually tailored temperature (pain levels of 50/100 based on pretests). The temperature remains constant over the next 120 s. Participants were asked to continuously record their pain level using a 10 cm Computerized Visual Analog Scale (CoVAS). Participants were asked to move the slider to reflect their pain from the left boundary (identified as “no pain”—score = 0) to the right boundary (identified as “worst pain imaginable”—score = 100). The CoVAS sampling rate was set at 10 Hz (10 pain measurements per second). The CS consists of a cold pressor test (CPT), wherein subjects immerse the opposite forearm in a cold-water bath (10°) for 120 s. The thermal stimulation intensities used before and after the music conditions are the same as the one used for the CPM paradigm.

### Measures

Sociodemographic data collected were sex, age and years of schooling.

For anxiety, the State Trait Anxiety Inventory (STAI) was used. There are two subscales: one for trait anxiety (STAI-Trait Y2) and one for state anxiety (STAI-State Y1). Each subscale contains 20 items and each statement is rated on a 4-point scale from: 1 “not at all” to 4 “very much so.” The overall score for each subscale ranges from 20 to 80. Participants with a score of 20–45 have low anxiety, 46–55 moderate anxiety and 56–80 severe anxiety.

For depression, the Beck Depression Inventory (BDI) was used. There are 21 items on a 4-point scale, so the overall score is from 0 to 63. Participants with a score from 0 to 10 do not have depression, between 11 and 16, they have mild mood disturbance, 17–20 borderline clinical depression, 21–30 moderate depression, 31–40 severe depression and more than 40 extreme depression.

For pain catastrophizing, the Pain Catastrophizing Scale (PCS) was used. There are 13 items from 0: “not at all” to 4: “all the time”. The overall score ranges from 0 to 52 in which participants with a score between 0 and 16 are non-catastrophizers, 17–29 are low catastrophizers and 30–52 severe catastrophizers.

At the end of the music intervention (i.e., after 20 min of listening), the perception of time was evaluated. The duration of the session was not communicated to the participants and they were asked how long they thought the session had lasted.

### Procedure

There was three testing days for each participant. The participant was seated in a comfortable chair in a quiet room. The first day, before the pain tests started, consent form was read and signed by the participants. Then, the MLM and LLM were determined according to the process described above. Sociodemographic data, STAI, BDI and PCS questionnaires were administered. Then, a first 2-min thermal pain test was performed followed by the CPT and a second thermal pain test. The second and third days consisted of a first two-minute thermal pain test followed by one of the music conditions (MLM or LLM). The order of music condition was randomized per a generated randomized sequence of integers based on a pseudo-random number algorithm. Under the music conditions, three thermal pain tests were performed: the first one at 2.20 min after the music started; the second one was after the relaxation phase at 11.30 min and the third one after the whole 20-min cycle. STAI questionnaire was also completed during these testing days.

### Statistical analyses

Based on data from previous studies managed in the Pain Research Laboratory (MUSEC: music, emotion and cognition), an effect size (d of Cohen) of 0.69 was used for sample size calculation (49). With a power of 80% and a type I risk of 5% (50), thirty-three participants needed to be included.

Evolution of overall pain perception was performed using a mixed effect model for repeated measures with an unstructured covariance matrix. Comparisons of continuous endpoints between pre-and post-condition, and between conditions were performed using paired student *t*-tests or Wilcoxon Sign Rank tests (non-parametric form of paired student *t*-tests, if distributions for variables were not normal). Bonferroni method was used for the correction of multiple comparisons.

All statistical tests were conducted using SAS® Studio (version 3.8, Edition enterprise, SAS Institute Inc, Cary, NC, USA). Comparisons of continuous endpoints between pre-and post-condition, and between conditions were performed using Wilcoxon Sign Rank tests (non-parametric form of paired student *t*-tests, as distributions for all dependent variables were not normal) (51). Normality of the distributions was tested using Shapiro-Wilk test. Comparisons between both music conditions were performed using Grizzle's model for crossover design with condition, period and sequence as fixed effects and participants within sequence as a random effect. Carryover effect was tested using Student *t*-tests. Statistics reported include means ± standard deviation and associated two-tailed *p* values as significance levels (cut-off of 0.05 for statistical significance). The research was submitted and approved by the Human Health Research Ethics Board from the CHUS. The analysis was conducted under the Intent-to-Treat (ITT) principle maintaining balance generated from the original random treatment allocation and avoiding statistical bias. As suggested for ITT, we ignored noncompliance, protocol deviations, withdrawal, and anything that happens after randomization (52). All subjects were then included in the analysis.

## Results

Thirty-three participants were included in the study. Three participants failed to complete the protocol (3 conditions), two completed only the CPT condition and one did not complete MLM condition. Among these 33 participants, all were randomized in the crossover part (17 to sequence 1 and 16 to sequence 2, Figure 1). The results of this controlled, randomized study are presented in compliance with the guidelines from the Consortium on the Assessment of Non-pharmacological Treatments (53).

The sociodemographic and baseline characteristics of the participants are described in Table 2. The mean age of the participants was 23.5 ± 4.9 years. There were 20 females (60.6%). In our sample, 28 participants had low trait anxiety, four moderate and one severe. For depression, 28 had no depression, four had mild mood disturbance and one had borderline clinical depression non-diagnosed. As for pain catastrophizing, we had 17 non-catastrophists, 14 mild catastrophizers and two severe ones.

**Table 2:** Demographic and baseline characteristics.

Characteristics	Total (*N* = 33)
Age (years), mean (SD)	23.5 (4.9)
Gender, *n* (%)	
Female	20 (60.6)
Male	13 (39.4)
STAI Y2 Trait Anxiety Score (20–80), mean (SD)	36.9 (10.7)
BDI score (0–63), mean (SD)	5.1 (4.6)
PCS score (0–52, mean (SD)	16.7 (10.2)

SD, standard deviation.

### Primary endpoint

**Figure 3:**
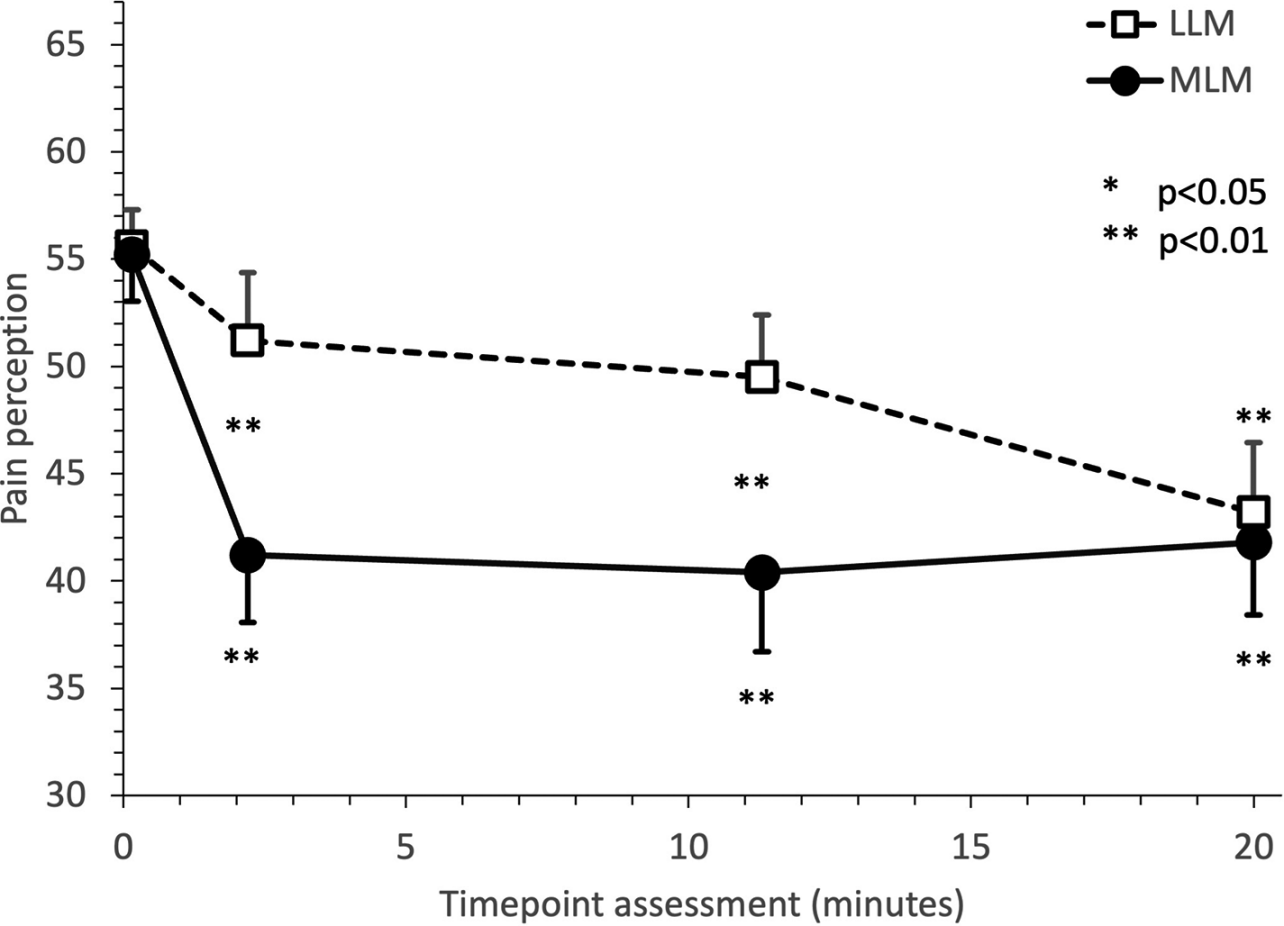
Evolution of mean pain perception under music intervention. Pain perception was collected using a Computerized Visual Analog Scale (CoVAS), which consists of a slider running along a 100 mm horizontal slider connected to the computer. Participants were asked to move the slider to reflect their pain from the left boundary (identified as “no pain”—score = 0) to the right boundary (identified as “worst pain imaginable”—score = 100). Timepoints of assessments were before music intervention, 2.20 min after the music started, 11.30 min after the music started (after the relaxation phase) at and after the whole 20-min cycle. Both conditions (LLM and MLM) are represented. Whiskers indicate SEMs.

Before the music intervention, mean pain perceptions were similar in both conditions: 55.2 (SD ± 12.0) in MLM and 55.6 (SD ± 9.5) in LLM. Overall, mean pain perceptions were significantly reduced under LLM and MLM conditions (*p* = 0.0090 and *p* < 0.0001, respectively), earlier under MLM condition (2.20 min) compared to LLM (20 min) (Table 3 and Figure 3).

**Table 3:** Pain perception under MLM and LLM.

Music	Timepoint	*n*	Value mean (SD)	Change mean (SD)	Reduction (%)	*p* value (pairwise)	*p* value (overall)
LLM	Before	31	55.6 (9.5)				
	2.20 min	31	51.2 (17.7)	−4.4 (14.2)	8	0.2862*	
	11.30 min	30	49.5 (15.8)	−5.7 (15.3)	10	0.1467*	
	20 min	31	43.2 (18.1)	−12.4 (18.4)	22	0.0024*	0.0090
MLM	Before	31	55.2 (12.0)				
	2.20 min	30	41.2 (17.2)	−14.2 (17.3)	26	0.0003*	
	11.30 min	30	40.4 (20.3)	−15.1 (17.5)	27	<0.0001*	
	20 min	30	41.8 (18.5)	−13.6 (18.0)	25	0.0009*	<0.0001
CPM	Before	33	59.2 (10.0)				
	After	33	44.5 (19.7)	−14.7 (29.9)	25	0.0003	

* Adjusted with Bonferroni correction.

Under LLM condition, the reduction in pain levels was 4.4 (SD ± 14.2) after 2.20 min (*p* = 0.2862), 5.7 (SD ± 15.3) after 11.30 min (*p* = 0.1467) and 12.4 (SD ± 18.4) after 20 min (*p* = 0.0024). Under MLM condition, the reduction in pain levels was 14.2 (SD ± 17.3) after 2.20 min (*p* = 0.0003), 15.1 (SD ± 17.5) after 11.30 min (*p* < 0.0001) and 13.6 (SD ± 18.0) after 20 min (*p* = 0.0009).

### Secondary endpoints

Reduction in pain observed at 2.20 min under MLM condition is comparable to the one observed under the CPM condition. Under CPM condition, the reduction in pain levels was 14.7 (SD ± 29.9) after the immersion of the participants’ arm in 10 degrees circulating water for 2 min.

Mean pain perceptions were significantly more reduced under MLM condition than under LLM condition at 2.20 min with an adjusted mean difference between both conditions of 9.7 (±3.7) (*p* = 0.0459) and at 11.30 min with an adjusted mean difference between both conditions of 8.9 (±3.4) (*p* = 0.0420) (Table 4). The differences between MLM and LLM are no longer significantly different after 20 min (*p* = 1.0000).

**Table 4:** Comparison of pain perception decrease between MLM and LLM.

Timepoint	Condition	LSMeans (SD)	95% CI	*p* value
2.20 min	MLM	−14.2 (2.8)	−20.0; −8.4	
	LLM	−4.5 (2.8)	−10.3; 1.2	
	LLM-MLM	9.7 (3.7)	2.0; 17.3	0.0459*
11.30 min	MLM	−15.1 (2.9)	−21.0; −9.2	
	LLM	−6.2 (2.9)	−12.1; −0.4	
	LLM-MLM	8.9 (3.4)	2.0; 15.8	0.0420*
20 min	MLM	−13.8 (3.1)	−20.2; −7.3	
	LLM	−12.4 (3.1)	−18.8; −6.1	
	LLM-MLM	1.3 (3.7)	−6.3; 8.9	1.0000*

Pain perception evolution is analysed using a generalized linear model with period, sequence and treatment as fixed effects, participants within sequence as random effect, and value before music intervention as a covariate.

* Adjusted with Bonferroni correction.

Regarding the perception of time, the musical intervention session appeared to be significantly shorter under MLM condition with a mean duration of 14.8 min (SD ± 5.5) than under the LLM condition with a mean perceived duration of 18.9 min (SD ± 7.1) (*p* = 0.0239).

No significant differences have been found between the conditions and the mood scores (BDI) or the pain catastrophizing scores (PCS). There was a small but significant level of anxiety difference between the 3 conditions (*p* = 0.0335), with an average value of 31.5 (±7.7) under the CPT condition, 30.7 (±9.4) under the LLM condition and 27.7 (±6.9) under the MLM condition.

Finally, to verify for similarities in the amplitude of the pain reduction between CPM and music sessions, correlation analysis were done between pain changes for CPM, MLM and LLM and different parameters like age and sex. A correlation on pain perception evolution at 2.20 min between CPM and LLM conditions was shown (Pearson coefficient of 0.36, *p* = 0.0471) as well as a correlation on pain perception evolution at 2.20 min between age and MLM condition (Pearson coefficient of 0.44, *p* = 0.0140) (Table 5).

**Table 5:** Influence of factors on pain perception decrease.

Factor	Timepoint	Condition	*p* value/*p* value (*r*)
Age		CPM	0.6896 (0.07)
	2.20 min	MLM	0.0140 (0.44)
	2.20 min	LLM	0.8306 (0.04)
	11.30 min	MLM	0.2943 (0.20)
	11.30 min	LLM	0.2332 (0.22)
	20 min	MLM	0.1327 (0.28)
	20 min	LLM	0.4922 (0.13)
Sex		CPM	0.5432
	2.20 min	MLM	0.4911
	2.20 min	LLM	0.8235
	11.30 min	MLM	1.0000
	11.30 min	LLM	0.6669
	20 min	MLM	0.3223
	20 min	LLM	0.6122
CPM	2.20 min	MLM	0.0546 (0.35)
	2.20 min	LLM	0.0471 (0.36)
	11.30 min	MLM	0.2409 (0.22)
	11.30 min	LLM	0.3285 (0.18)
	20 min	MLM	0.5043 (0.13)
	20 min	LLM	0.7403 (−0.06)

For factors age and CPM, r Pearson coefficient and *p* value are provided; for sex *p* value coming from Wilcoxon signed rank test is provided.

## Discussion

For the past few years, digital therapeutics (DTx), a subset of digital health, is changing the healthcare delivery system with evidence-based technologies driven by high quality software to prevent, manage, or treat a medical disorder or disease and that improve patient outcomes (54). The consensus among researchers in the field of DTx is that it requires more clinical data and investigation to be fully evaluated. Music is one of these approaches that was demonstrated to have significant pain reduction effects for different clinical conditions (3–6).

Several mechanisms have been suggested to understand music-induced pain reduction. A significant correlation between music pleasantness and pain reduction in healthy subjects was reported, suggesting the importance of the emotional valence for music-induced pain reduction (31). However, in another study also using experimental pain, the authors found that emotional responses were not correlated to the analgesic effects, but that perception of control in the selected music during the experiment and the engagement with music in the subject's everyday life were the most important parameters (55). Interestingly, antagonists drugs of endogenous dopamine and opioids did not reduce the effect of music analgesia (56). The authors found that the main source of the effect was related to the expectation of analgesia from music, suggesting mechanisms comparable to placebo analgesia. Based on these results, we could conclude that distraction and expectation are probably the main effect, but other mechanisms including endogenous pain modulation such as CPM was suggested (29). In support of this hypothesis, a study measured pain-related activity in the brain, brainstem, and spinal cord using magnetic resonance imaging (MRI) during sessions of favorite music versus no music (30). They found significant activation during the music session in regions related to descending pain inhibition mechanisms implicated in CPM such as the periaqueductal gray, rostral ventromedial medulla, and the spinal cord.

The goal of the present study was to compare the effect of most-liked music to least-liked music on pain perception at different times during the 20-min music sessions. We also compared the effects of CPM with the two music sessions on pain perception in the same subjects. A positive correlation between CPM and the music conditions will not determine if the mechanisms are the same but will give a hint in that direction and confirm the interest of future tests on music-induced analgesia mechanisms.

For the effect of music on pain, we found that the pain level decreased is significantly higher under MLM condition than under LLM condition from 2.20 min of listening to 11.30 min. At the end of the 20-min session, the decrease in pain level is comparable under LLM and MLM. Pain alleviation is thus faster and stable for MLM from the beginning up to 20 min, while the LLM's pain alleviation is by increments needing more than half of the session to perceive a decrease in pain with end results statistically comparable for both conditions.

The cold pressor pain significantly reduced pain perception, supporting a CPM effect. The only significant correlation between music and CPM is for pain perception at 2.20 min for LLM. We can theorize that the “unpleasant” effect of the least preferred music might have activated inhibitory mechanisms such as the unpleasant aspect of the cold pressor pain during the immersion of the arm in cold water. We did not systematically ask for feedback regarding the music at the end of the session, but several subjects spontaneously reported that they finally learned to enjoy the music that they rated as their LLM at the beginning later during the listening. As the music ends up being enjoyed, this “counter-irritation-like” effect of “unpleasant music” seems to disappear with time. This could be due to the mere-exposure effect (57). This cognitive principle states that the more you are exposed to something, the more you like it. This could also suggest that the mechanism of action of music could be comparable to CPM over the first 2 min, but that beyond that, music would allow pain control according to other neurophysiological mechanisms.

It was reported that pleasant music decreased pain more than unpleasant music and silence (31). This is congruent in part with our results as after 2.20 min of LLM, pain perception was higher than after MLM. Participants reported enjoying the MLM more than the LLM when choosing the music at the beginning. Some subjects also spontaneously reported learning to enjoy fairly more the LLM over time. All the conditions may act from different endogenous descending modulatory systems, according to the time frames. More direct evaluation of the implicated mechanisms would be of interest in future studies.

Another interesting aspect is the perception of time during the music conditions. The musical intervention session appeared to be perceived as significantly shorter under MLM condition than the LLM condition. This effect could be related to the “immersive” effect of most-liked music compared to least-liked music. Using subjects’ selected music in video games is enhancing time underestimation (58). Other important psychological factors are anxiety, depression and pain catastrophizing that can affect pain perception (59–61). In this study the only significant effect was a lower anxiety score during MLM compared to CPT, suggesting a relaxation effect of MLM. Altogether these results suggest that MLM is rapidly active in reducing pain, reduce anxiety and give the impression that the time was shorter than it was. All positive characteristics for intervention pain control during painful procedures.

MUSIC CARE©, the web app-based music intervention evaluated has good ecological validity, which is hard to find in experimental music studies. It is already used in clinical contexts to alleviate pain and allows for reduced consumption of analgesics. The analgesia provided by medication usually starts 20–30 min after intake. However, the MLM chosen shows here a music-induced analgesia already present after 2.20 min and maintained for 20 min. As a result, using the participants’ favorite MUSIC CARE©'s style could be a convenient and valuable adjuvant to acute pain treatments. Moreover, with its selection of 30 different styles of music, it has more potential of personalized care. Patients in clinics could also bring their own music and increase even more the valence of the music and its associated analgesia. Nevertheless, in an experimental setting, MUSIC CARE© allowed for a higher comparability between the music sequences compared to many music interventions studies as they are all constructed in the same way.

This study has some limitations. We compared music-induced analgesia to the cold bath to induce CPM to look for similarities or differences in responses. Other control conditions could have been used. The silence gives a setting with no distraction of attention or emotions (62). White noise, pink noise (white noise using the same frequency range as music) or audio books could be used. These approaches distract attention and have very limited emotional potential (63). They are painless auditory inputs and already used in some research as control conditions to music (11). Future studies comparing different distraction modalities could be of interest.

These results are comparable to previous studies (64–66). Future studies with patients experiencing chronic pain to see the effect on clinical pain and related endogenous pain modulation mechanisms would be important. Brain activation could be assessed as well when listening to MUSIC CARE© to better understand the related brain regions implicated. To further explore musical appreciation, it would also have been interesting to include any measures that extend beyond participants’ mentions of their own experiences with the conditions. The literature supports enormous diversity in antecedents and causes of music appreciation across contexts, individuals, cultures, and historical periods. But the processes implicated in that are still unexplored (67).

In conclusion, MLM significantly reduce pain perception and more rapidly than LLM, but both types are analgesics after 20 min. Interestingly, the LLM pain reduction was correlated with CPM after 2.20 min. We hypothesized that the unpleasantness of the LLM music is triggering a “counterirritation effect” possibly comparable to CPM that fades over time when the unpleasantness seems to fade over time. LLM is correlated with CPM but not MML, suggesting different mechanisms between LLM and MLM.

In France, general practitioners can now prescribe music as part of the overall pain management of patients suffering from chronic diseases. This means that apps like MUSIC CARE© can be prescribed by general practitioners and used outside the hospital environment. The MUSIC CARE© application is currently used in 500 hospital departments around the world. The music intervention is administered *via* a smartphone- (or tablet- and computer-) based application called MUSIC CARE© which is low-cost, highly available to the public, and usable in a home environment. The MUSIC CARE© app is a receptive music intervention, allowing the patient to freely adjust the length of and choose the preferred style between varying sequences of instrumental music.

## Data Availability

The raw data supporting the conclusions of this article will be made available by the authors, without undue reservation.
